# Partial Amniote Sex Chromosomal Linkage Homologies Shared on Snake W Sex Chromosomes Support the Ancestral Super-Sex Chromosome Evolution in Amniotes

**DOI:** 10.3389/fgene.2020.00948

**Published:** 2020-08-18

**Authors:** Worapong Singchat, Syed Farhan Ahmad, Siwapech Sillapaprayoon, Narongrit Muangmai, Prateep Duengkae, Surin Peyachoknagul, Rebecca E. O’Connor, Darren K. Griffin, Kornsorn Srikulnath

**Affiliations:** ^1^Laboratory of Animal Cytogenetics and Comparative Genomics (ACCG), Department of Genetics, Faculty of Science, Kasetsart University, Bangkok, Thailand; ^2^Special Research Unit for Wildlife Genomics (SRUWG), Department of Forest Biology, Faculty of Forestry, Kasetsart University, Bangkok, Thailand; ^3^Department of Fishery Biology, Faculty of Fisheries, Kasetsart University, Bangkok, Thailand; ^4^School of Biosciences, University of Kent, Canterbury, United Kingdom; ^5^Center for Advanced Studies in Tropical Natural Resources, National Research University-Kasetsart University, Kasetsart University, Bangkok, Thailand; ^6^Center of Excellence on Agricultural Biotechnology (AG-BIO/PERDO-CHE), Bangkok, Thailand; ^7^Omics Center for Agriculture, Bioresources, Food and Health, Kasetsart University (OmiKU), Bangkok, Thailand; ^8^Amphibian Research Center, Hiroshima University, Higashihiroshima, Japan

**Keywords:** chromosome map, snake, BAC, retrotransposon, super-sex chromosome

## Abstract

Squamate reptile chromosome 2 (SR2) is thought to be an important remnant of an ancestral amniote super-sex chromosome, but a recent study showed that the Siamese cobra W sex chromosome is also a part of this larger ancestral chromosome. To confirm the existence of an ancestral amniote super-sex chromosome and understand the mechanisms of amniote sex chromosome evolution, chromosome maps of two snake species [Russell’s viper: *Daboia russelii* (DRU) and the common tiger snake: *Notechis scutatus* (NSC)] were constructed using bacterial artificial chromosomes (BACs) derived from chicken and zebra finch libraries containing amniote sex chromosomal linkages. Sixteen BACs were mapped on the W sex chromosome of DRU and/or NSC, suggesting that these BACs contained a common genomic region shared with the W sex chromosome of these snakes. Two of the sixteen BACs were co-localized to DRU2 and NSC2, corresponding to SR2. Prediction of genomic content from all BACs mapped on snake W sex chromosomes revealed a large proportion of long interspersed nuclear element (LINE) and short interspersed nuclear element (SINE) retrotransposons. These results led us to predict that amplification of LINE and SINE may have occurred on snake W chromosomes during evolution. Genome compartmentalization, such as transposon amplification, might be the key factor influencing chromosome structure and differentiation. Multiple sequence alignments of all BACs mapped on snake W sex chromosomes did not reveal common sequences. Our findings indicate that the SR2 and snake W sex chromosomes may have been part of a larger ancestral amniote super-sex chromosome, and support the view of sex chromosome evolution as a colorful myriad of situations and trajectories in which many diverse processes are in action.

## Introduction

Observations suggest that the common ancestor of snakes and birds lived about 260 million years ago, with gross chromosome morphology diverging soon after (Smith and Sinclair, 2004; Vallender and Lahn, 2006). Snakes possess a ZZ male/ZW female sex chromosome system, in which the snake Z sex chromosome is similar in size to that of most birds (Beçak et al., 1964; Bianchi et al., 1969; Vallender and Lahn, 2006; Ellegren, 2010). Ohno (1967) hypothesized that birds and snakes share a ZZ male/ZW female sex chromosome system, however, recent comparisons of chromosome mapping and genome sequence analyses revealed completely different linkage homologies between snake and bird Z chromosomes (Matsuda et al., 2005; Matsubara et al., 2006, 2012; Srikulnath et al., 2009a,b, 2013, 2014, 2015; Uno et al., 2012; Singchat et al., 2018, 2020). That is, the chicken Z sex chromosome is homologous to parts of chromosome 2p in most snakes and also in other squamate reptiles, whereas the snake Z sex chromosome is homologous to the chicken chromosomes 2p and 27. These conserved linkage homologies are shared across most snake species studied (Matsuda et al., 2005; Matsubara et al., 2006, 2012; Srikulnath et al., 2009a,b, 2013, 2014, 2015; Ezaz et al., 2017; Laopichienpong et al., 2017a,b; Tawichasri et al., 2017; Singchat et al., 2018, 2020), whereas two henophidian snakes (primitive snakes), python (*Python bivittatus*), and boid (*Boa imperator*), are thought to have XX/XY sex-determination systems (Gamble et al., 2017). Interestingly, sex chromosomal linkage homologies have also been found between chromosomes in different amniote lineages, and unrelated sex chromosomes share linkage homologies across distantly related groups (Ezaz et al., 2017). To understand the phenomenon of unrelated sex chromosomal linkages, specifically those of snakes, a number of molecular resources for comparative genomic analyses have been developed for the Siamese cobra (*Naja kaouthia*). These include sex-linked markers, a bacterial artificial chromosome (BAC) chromosome map, and genome-wide single nucleotide polymorphic analysis (Singchat et al., 2018, 2020; Laopichienpong et al., submitted data). Together, these findings suggest that in addition to squamate reptile chromosome 2 (SR2), the Siamese cobra W sex chromosomes share partial sex chromosomal linkage homologies with sex-related elements of other amniotes, despite their apparent diversity of sex determining mechanisms (Ezaz et al., 2017; Singchat et al., 2018, 2020; Matsubara et al., 2019; Ahmad et al., 2020).

Snakes possess highly conserved karyotypes with chromosome numbers mostly around 2n = 36, but nonetheless significant variation (2n = 24–50) (Beçak et al., 1964; Beçak and Beçak, 1969; Matsuda et al., 2005; Matsubara et al., 2006, 2012, 2015a; Oguiura et al., 2009; Singchat et al., 2018, 2020; Thongchum et al., 2019). Snake karyotypes are characterized as “bimodal,” comprising macro- and microchromosomes, the latter defined as nearly indistinguishable by shape and centromere position due to their small size (Olmo, 2008; Srikulnath et al., 2015). The Z sex chromosome, which is similar in size across species, is the fourth or fifth largest metacentric chromosome, whereas the W sex chromosome varies from being homomorphic with the Z in most henophidians to highly differentiated in advanced snakes, as a consequence of different centromere positions and/or amounts of heterochromatin (Matsubara et al., 2006, 2012; O’Meally et al., 2010; Augstenová et al., 2018; Singchat et al., 2018, 2020). Progressive stages in sex chromosome degeneration have been observed among snake lineages (Matsubara et al., 2006, 2012, 2015a, 2016a; Laopichienpong et al., 2017a,b; Augstenová et al., 2018; Singchat et al., 2018). Chromosomal rearrangements are considered to influence the organization and function of genomic elements such as gene expression, changing regulatory elements, and position effects (Chadov et al., 2004; Harewood and Fraser, 2014; Stewart and Rogers, 2019). Variations in snake diploid chromosome numbers and/or structure of the W chromosome are likely to be related to the diversity of snake species (Olmo and Signorino, 2005; Oguiura et al., 2009), given that sex chromosomes are probably affected by postzygotic isolation mechanisms among divergent snake lineages, including lineages with either cryptic or advanced W sex chromosomes (Organ and Janes, 2008; Sabath et al., 2016).

A complex chromosomal structure is always observed on the heteromorphic W sex chromosome of advanced snakes in that large amplification of satellite DNA, microsatellite repeat motifs, and telomeric repeats have been found (O’Meally et al., 2010; Matsubara et al., 2016b; Augstenová et al., 2018; Singchat et al., 2018; Thongchum et al., 2019). Remarkably, non-homologous W sex chromosomes of the chicken and the common tiger snake [*Notechis scutatus* (NSC)] share frequent repeat sequences that are not present elsewhere in the genomes. Chromosome mapping of the intronic sequences of doublesex and mab-3 related transcription factor 1 (*DMRT1*) and *CTNNB1* (encoding β-catenin) as sex-linked genes in birds and snakes, respectively, has revealed massive amplification in discrete domains on the W sex chromosome of the common tiger snake (O’Meally et al., 2010). Collectively, this suggests that common genomic regions such as repeats are partially shared between chicken and snake sex chromosomes, supporting the hypothesis that SR2 and snake W sex chromosome are associated with a larger ancestral amniote super-sex chromosome (Ezaz et al., 2017; Singchat et al., 2018, 2020; Matsubara et al., 2019; Ahmad et al., 2020). Although this hypothesis has been reasonably well studied, a large information gap still exists in terms of the molecular characteristics that would allow a better understanding of the complex structure and homology of snake W sex chromosomes. If such remarkable diversity and evolutionary activity are possible within this small clade of larger snake lineages such as the Siamese cobra or the common tiger snake (O’Meally et al., 2010; Matsubara et al., 2016b; Singchat et al., 2018; Thongchum et al., 2019), additional model clades are required to determine whether there may be any scientific interest in this area regarding other large snakes. Therefore, it is worth investigating the evolutionary context of unrelated sex chromosomal linkage homologies in additional snake species to gain novel insights pertaining to the possible existence of an ancestral amniote super-sex chromosome. Taking this scenario together with a map of the Siamese cobra chromosome presented by Singchat et al. (2018), we propose two hypotheses: (i) there is a shared unrelated sex chromosomal linkage homology among amniotes in other snakes, and (ii) common genomic segments/elements are shared among unrelated sex chromosomal linkage homologies of amniotes.

To address these hypotheses, we constructed a comparative chromosome map, using 47 BACs derived from chicken [*Gallus gallus* (GGA)] and zebra finch [*Taeniopygia guttata* (TGU)] genomes using fluorescence *in situ* hybridization (FISH) in combination with our previously published data in relation to sex chromosomal linkage homologies in amniotes (Damas et al., 2017; O’Connor et al., 2018a,b; Singchat et al., 2018, 2020). These BAC chromosome maps also allowed us to detect changes in chromosomal positions when the order of BACs is modified due to chromosomal rearrangements. We compared the sex chromosomal linkage homologies of Russell’s viper [*Daboia russelii* (DRU), Viperidae] and the common tiger snake (NSC, Elapidae) with those of the Siamese cobra [*N. kaouthia* (NKA), Elapidae] and other amniotes. We then determined the *in silico* proportion of repeats on the chicken and zebra finch BAC sequences for comparison as the potential driving force for sex chromosome differentiation in future testing. In the light of these findings, we provide a comparative genomic overview, revealing the shared sex chromosomal linkage homology between snakes and amniotes. Our results allow an improved understanding of the transition mechanisms between different sex chromosome forms among amniotes. We propose the possibility of shared unrelated linkage homologies through an ancestral super-sex chromosome.

## Materials and Methods

### Specimen Collection and Chromosome Preparation

Cell suspensions of two female species [Russell’s viper (DRU) and the common tiger snake (NSC)] were provided by Malcolm Ferguson-Smith (Department of Veterinary Medicine, University of Cambridge, United Kingdom) in November 2019. The cells were dropped onto clean glass slides and air-dried. The slides were stored at −80°C until subsequent analysis.

### Karyotyping

The morphology and size of macrochromosomes were characterized and arranged in accordance with Levan et al. (1964) and Turpin and Lejeune (1965).

### Isolation, Amplification, and Labeling of Chicken and Zebra Finch BACs

Chicken and zebra finch BACs were applied for cross-species FISH mapping based on the range of the proportion of conserved elements shared across multiple species. Given the high degree of apparent genome conservation between avian and reptilian species (Damas et al., 2017; O’Connor et al., 2018a,b; Singchat et al., 2018, 2020), these sets of BACs were applied to DRU and NSC. In total, 32 chicken and 15 zebra finch BACs were anchored to chicken and zebra finch genome assemblies by linkage and sequencing comprising chicken chromosome 1 (GGA1), GGA2p, GGA4p, GGA5, GGA6, GGA9, GGA13, GGA15, GGA17, GGA23, GGA27, GGA28, and chicken sex chromosome Z (GGAZ), and zebra finch chromosome 1 (TGU1B), TGU4A, TGU5, TGU6, TGU9, TGU13, TGU15, TGU17, TGU23, TGU27, TGU28, and TGUZ. The BAC clone DNA was isolated using the Qiagen Miniprep Kit (Qiagen, Manchester, United Kingdom) prior to amplification and direct labeling by nick translation (Roche, Welwyn Garden City, United Kingdom). Probes were labeled with Texas Red 12-dUTP (Invitrogen Corporation and Applied Biosystems Inc., Carlsbad, CA, United States) and fluorescein isothiocyanate (FITC)-12-UTP (Roche) prior to purification using the Qiagen Nucleotide Removal Kit (Qiagen).

### Cross-Species BAC FISH Mapping

Chromosome slides were dehydrated through an ethanol series (2 min each in 2 × SSC, 70, 85, and 100% ethanol at room temperature). Probes were diluted in a hybridization solution (Cytocell Ltd., Cambridge, United Kingdom) with chicken hybloc (Insight Biotechnology Ltd., London, United Kingdom) and applied to DRU and NSC chromosomes on a 37°C hotplate before being sealed with rubber cement. Probe and target DNA were simultaneously denatured on a 75°C hotplate prior to hybridization for 2 min in a humidified chamber at 37°C for 72 h. Slides were washed post-hybridization for 30 s in 2 × SSC/0.05% Tween 20 at room temperature before being counterstained using VECTASHIELD antifade mounting medium with 4′,6-diamidino-2-phenylindole (DAPI; Vector Laboratories, Inc., Burlingame, CA, United States). Images were captured using the Olympus BX61 epifluorescence microscope with a cooled CCD camera and SmartCapture system (Digital Scientific UK Ltd., Cambridge, United Kingdom). Confirmation of BAC signal order was achieved by dual-color experiments, where Texas Red 12-dUTP- and FITC-12-UTP-labeled probes were hybridized simultaneously.

### BAC Sequence Analysis

In addition to gene position, gene structure, and microsatellite repeat motifs in each BAC as identified in our previous study (Singchat et al., 2018, 2020), the abundance of repeat elements were annotated using RepeatMasker 4.0.3 (Smit et al., 2013). Sequences were aligned against the crossmatch search engine and the reference species was set as “chicken.” The output files were parsed with custom bash scripts and results were plotted as a bar graph. Boxplots showing averages of repeat percentages between BACs mapped on autosomes of snake and BACs mapped on snake W sex chromosomes were performed using “boxplot” in R statistical software version 3.4.4 (R Core Team, 2019). Data are shown as mean ± standard deviation. The box includes 50% of the data, and the whiskers reach the highest and lowest values within 95% of the distribution. Open circles represent single values outside 95% of the distribution. Differences in averages of repeat percentages between BACs mapped on autosomes of snake and BACs mapped on snake W sex chromosomes were determined by *t*-test. Degrees of statistical significance were represented as ^∗^ for *p* ≤ 0.05, ^∗∗^ for *p* ≤ 0.01, and ^∗∗∗^ for *p* ≤ 0.001. To detect any possible shared conserved elements, all candidate BAC sequences were aligned against each other using multiple sequence alignment (MSA) tools: ClustalW (Larkin et al., 2007) and Muscle (Edgar, 2004) to predict any conserved sequences. All combinations of multiple alignments were compared and output MSA fasta files were visualized using Geneious software (version 11.1.4)^1^. These sequences were then used to search in the Ensembl Genome Browser^2^ against the amniote database using the Basic Local Alignment Search Tool program for their homologs among different groups (Cunningham et al., 2019). Each sequence was mapped *in silico* on chromosomes under the available genome using the chromosome scale in the Ensembl database.

## Results

### Chromosome Constitution and Sex Chromosomal Linkage Homology of Snakes

**FIGURE 1:**
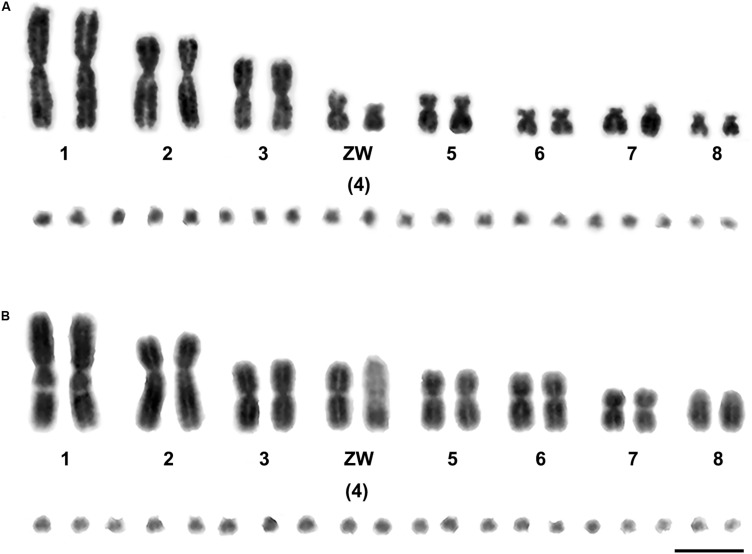
Gray images of DAPI-stained karyotypes of Russell’s viper (*Daboia russelii*) **(A)** and the common tiger snake (*Notechis scutatus*) **(B)**. Scale bar represents 10 μm.

We examined more than 20 DAPI-stained metaphase spreads for one female Russell’s viper (DRU) and one female Tiger snake (NSC). The diploid chromosome numbers were 36, comprising eight pairs of macrochromosomes and 10 pairs of microchromosomes in each species. The eight pairs of macrochromosomes of DRU and NSC comprised two pairs of large metacentric (1st and 3rd) and one pair of submetacentric chromosomes (2nd), two pairs of medium-sized metacentric chromosomes (5th–6th), two pairs of small submetacentric chromosomes (7th–8th), and 10 pairs of microchromosomes (9th–18th). The fourth largest chromosomes were sex chromosomes, in which the female karyotype contained the metacentric Z chromosome, the submetacentric W chromosome for DRU, and the large acrocentric W chromosome for NSC (Figure 1).

**FIGURE 2:**
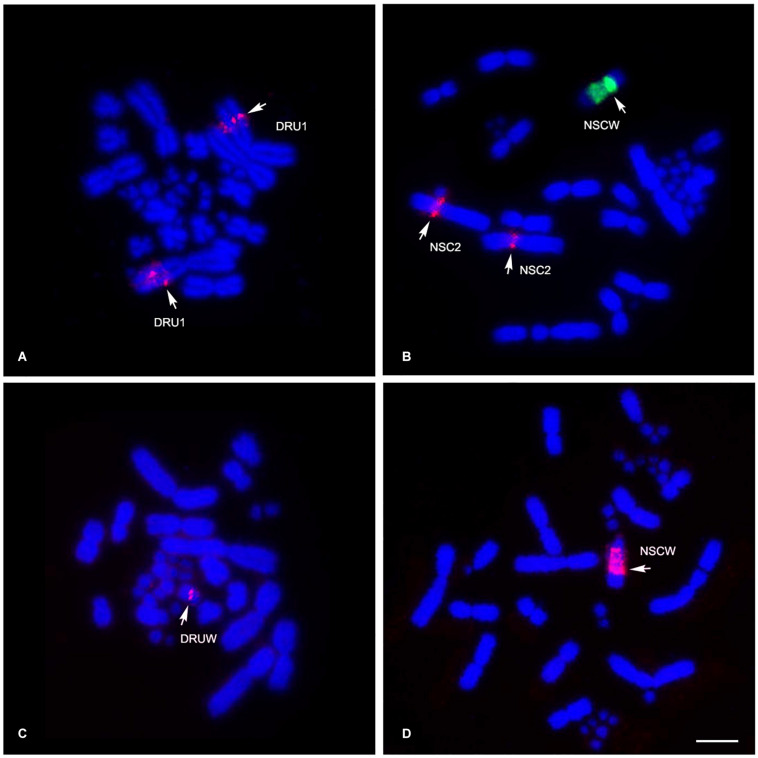
Chromosomal locations of chicken and zebra finch BACs in Russell’s viper (*Daboia russelii*) and the common tiger snake (*Notechis scutatus*). Russell’s viper, GGA5 BAC [Texas Red-labeled CH261-122F8 (red)] was localized to chromosome 1 (DRU1) **(A)** and GGAZ BAC [Texas Red-labeled TGMCBA-270I9 (red)] was localized to W sex chromosome (DRUW) **(C)**. Common tiger snake, GGA1 BAC [Texas Red-labeled CH261-125F1 (red) and fluorescein isothiocyanate-labeled TGMCBA-167P13 (green)] were localized to chromosome 2 (NSC2) and W sex chromosome (NSCW), respectively **(B)** and GGAZ BAC [Texas Red-labeled TGMCBA-270I9 (red)] was localized to W sex chromosome (NSCW) **(D)**. Arrows indicate hybridization signals. Scale bar represents 10 μm.

**FIGURE 3:**
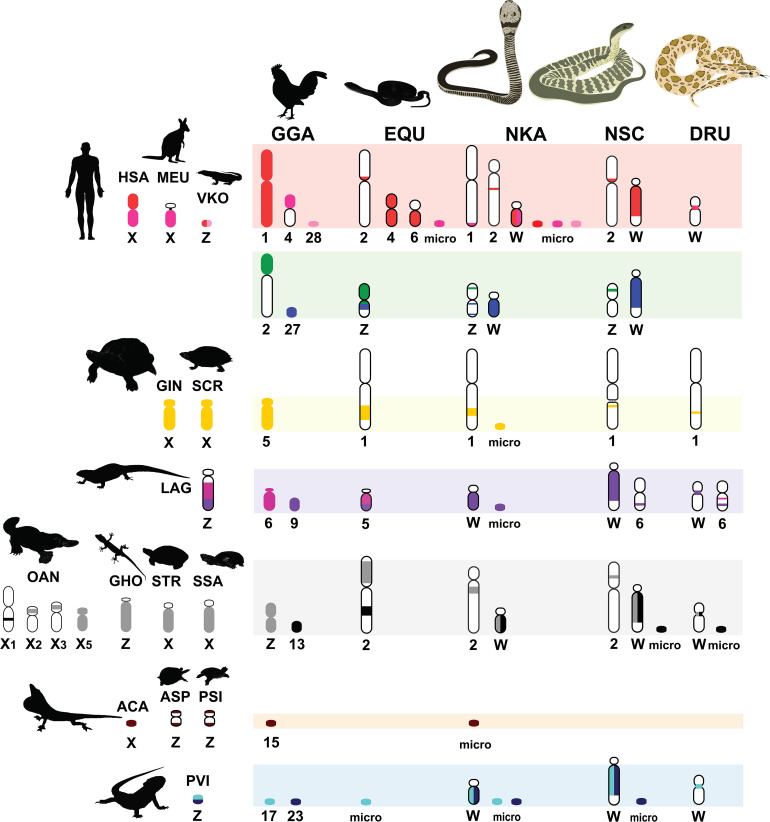
Comparative chromosome maps of chromosomes among the Siamese cobra (*Naja kaouthia*, NKA), Russell’s viper (*Daboia russelii*, DRU), and the common tiger snake (*Notechis scutatus*, NSC). Maps were constructed with 47 BACs and linkage maps of chicken (*Gallus gallus*, GGA), Japanese four-striped rat snake (*Elaphe quadrivirgata*, EQU), and chromosomes sharing homologies with sex chromosome of several other amniotes. Chromosome numbers indicate chicken (GGA), humans (*Homo sapiens*, HSA), tammar wallaby (*Macropus eugenii*, MEU), duck-billed platypus (*Ornithorhynchus anatinus*, OAN), green anole (*Anolis carolinensis*, ACA), bearded dragon lizard (*Pogona vitticeps*, PVI), sand lizard (*Lacerta agilis*, LAG), Hokou gecko (*Gekko hokouensis*, GHO), Komodo dragon (*Varanus komodoensis*, VKO), marsh turtle (*Siebenrockiella crassicollis*, SCR), wood turtle (*Glyptemys insculpta*, GIN), Mexican musk turtle (*Staurotypus triporcatus*, STR), giant musk turtle (*Staurotypus salvinii*, SSA), spiny softshell turtle (*Apalone spinifera*, ASP), and Chinese softshell turtle (*Pelodiscus sinensis*, PSI), showing partial homologies with Siamese cobra, green iguana (*Iguana iguana*), common garden lizard (*Calotes versicolor*), and water monitor lizard (*Varanus salvator macromaculatus*) chromosomes. Partial sex chromosomal linkage homologies are shown in the same color. Chromosomal locations of genes in the amniotes were obtained from comparative gene mapping (chromosome mapping via a cytogenetic technique) and whole genome sequencing as the following sources: GGA from Matsuda et al. (2005), HSA and MEU from Grützner et al. (2004), OAN from Veyrunes et al. (2008), ACA from Alföldi et al. (2011), PVI from Ezaz et al. (2013), LAG from Srikulnath et al. (2014), GHO from Kawai et al. (2009), VKO from Lind et al. (2019), EQU from Matsubara et al. (2006), SCR from Kawagoshi et al. (2012), GIN and STR from Montiel et al. (2016), SSA from Kawagoshi et al. (2014), ASP from Badenhorst et al. (2013), and PSI from Kawagoshi et al. (2009).

Different degrees of cross-species BAC hybridization reflect how conserved segments have changed during evolution (Griffin et al., 2008; Skinner et al., 2009; Lithgow et al., 2014; Damas et al., 2017; O’Connor et al., 2018a,b; Singchat et al., 2018, 2020). Here, chicken and zebra finch BACs located on GGA1, GGA2p, GGA4p, GGA5, GGA6, GGA9, GGA13, GGA15, GGA17, GGA23, GGA27, GGA28, and GGAZ were mapped to DRU (six chicken BACs and eight zebra finch BACs) and NSC (9 chicken BACs and 10 zebra finch BACs) (Figures 2,3 and Table 1). More than 20 metaphase spreads were observed for each BAC, with hybridization efficiencies ranging approximately from 70 to 90%. Chromosome homology among DRU and NSC, chicken, and zebra finch was analyzed using the chicken genome database^3^ and the zebra finch genome database^4^. In DRU chromosome mapping, one BAC mapped on DRU1 was homologous to GGA5 (CH261-122F8), one BAC mapped on DRU3 was homologous to GGA2q (CH261-44D16), and two BACs mapped to DRU6 were localized to GGA6 (CH261-67H5) and GGA9 (CH261-95N3). One BAC mapped on one pair of microchromosomes was localized to GGA13 (TGMCBA-321B13). Interestingly, an additional seven BACs previously mapped on the W sex chromosome of NKA (NKAW) (Singchat et al., 2018) were also mapped on the W sex chromosome of DRU (DRUW), and were homologous to GGA4 (CH261-71L6), GGA9 (CH261-95N3), TGU1B (TGMCBA-167P13), TGU9 (TGMCBA-217A3), TGU13 (TGMCBA-136I12), and TGU17 (TGMCBA-375I5 and TGMCBA-67H23). Moreover, three BACs mapped on DRUW were located on GGA1 (CH261-98G4), GGA9 (TGMCBA-321L6), and GGAZ (TGMCBA-270I9) (Figure 2 and Table 1).

**TABLE 1:** Chicken and zebra finch BACs mapped on Siamese cobra (*Naja kaouthia*, NKA), Russell’s viper (*Daboia russelii*, DRU), and common tiger snake (*Notechis scutatus*, NSC) chromosomes and their chromosomal locations in chicken (*Gallus gallus*, GGA).

**Name**	**GGA**	**Result**		
		**NKA**	**NSC**	**DRU**
CH261-125F1	1	2q	2	–
TGMCBA-167P13	1	Wq	W	W
CH261-184E5	1	micro	–	–
CH261-18J16	1	micro	–	–
CH261-36B5	1	micro	–	–
CH261-83O13	1	micro	–	–
CH261-89C18	1p	Wq	–	–
CH261-98G4	1q	–	–	W
CH261-123O22	2	–	Z	–
CH261-177K1	2	Z	–	–
CH261-44D16	2q	–	3	3
CH261-18C6	4	1q and micro	–	–
CH261-71L6	4	Wq	–	W
CH261-122F8	5	–	1q	1q
CH261-2I23	5	1q	–	–
CH261-49B22	5p	1q and micro	–	–
TGMCBA-145C6	5	1q	–	–
CH261-67H5	6	–	–	6
CH261-183N19	9p	micro	–	–
CH261-187M16	9q	micro	–	–
CH261-95N3	9	Wq	6	6, W
TGMCBA-217A3	9	Wq	W	W
TGMCBA-321L6	9	–	–	W
TGMCBA-136I12	13	Wq	W	W
TGMCBA-321B13	13q	–	micro	micro
CH261-40D6	15	micro	micro	–
CH261-48M1	15	–	micro	–
CH261-90P23	15p	micro	–	–
TGMCBA-266G23	15q	micro	–	–
CH261-69M11	17	micro	–	–
TGMCBA-375I5	17p	Wq	W	W
TGMCBA-67H23	17	Wq	W	W
CH261-105P1	23	micro	–	–
CH261-191G17	23p	micro	–	–
CH261-49G9	23	micro	–	–
CH261-90K11	23q	–	micro	–
TGMCBA-173N15	23	micro	–	–
TGMCBA-227A15	23	Wq	W	–
TGMCBA-48O8	23	micro	–	–
CH261-66M16	27p	Z	–	–
TGMCBA-23C5	27	Zq and Wq	W	–
CH261-64A15	28p	micro	–	–
CH261-72A10	28q	micro	–	–
CH261-129A16	Zp	Wq	–	–
CH261-133M4	Zq	2q	2p, W	–
TGMCBA-200J22	Z	Wq	W	–
TGMCBA-270I9	Z	2q	W	W

*–, No signal.*

For NSC chromosome mapping, one BAC mapped on NSC1 was homologous to GGA5 (CH261-122F8), two BACs on NSC2 to GGA1 (CH261-125F1) and GGAZ (CH261-133M4). One BAC mapped on NSC3 was localized to GGA2q (CH261-44D16), one BAC on NSCZ to GGA2p (CH261-123O22), and one BAC on NSC6 to GGA9 (CH261-95N3). Four BACs mapped on different pairs of microchromosomes were localized to GGA13 (TGMCBA-321B13), GGA15 (CH261-40D6 and CH261-48M1), and GGA23 (CH261-90K11), respectively. Remarkably, an additional eight BACs previously mapped on NKAW (Singchat et al., 2018) were also mapped on the W sex chromosome of NSC (NSCW), which had homology with TGU1B (TGMCBA-167P13), TGU9 (TGMCBA-217A3), TGU13 (TGMCBA-136I12), TGU17 (TGMCBA-375I5 and TGMCBA-67H23), TGU23 (TGMCBA-227A15), TGU27 (TGMCBA-23C5), and TGUZ (TGMCBA-200J22). Moreover, two BACs mapped on NSCW were located on GGAZ (CH261-133M4 and TGMCBA-270I9) (Figure 2 and Table 1).

### *In silico* Searching and Mapping of BACs in Amniote Genomes

**FIGURE 4:**
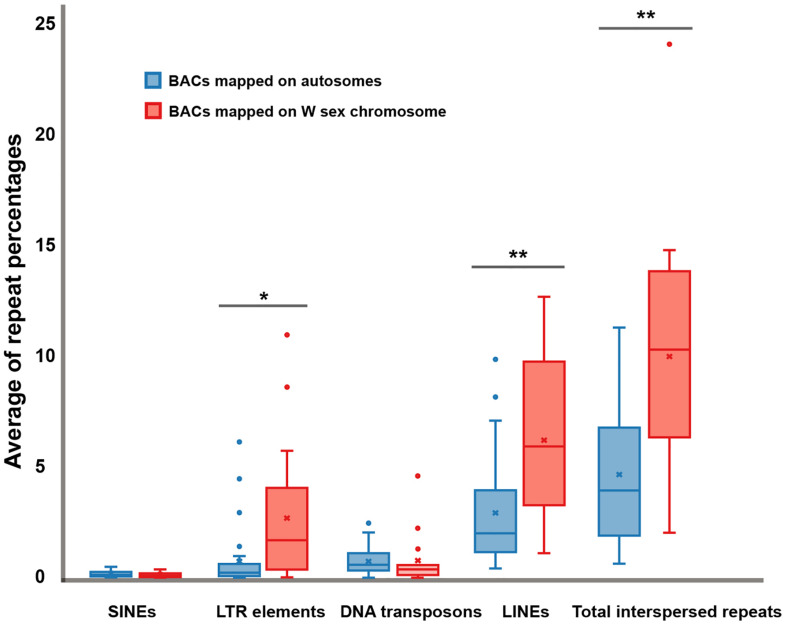
Boxplots showing average of repeat percentages between BACs mapped on autosomes of snake (blue) and BACs mapped on snake W sex chromosomes (red) of three snake species: the Siamese cobra (*Naja kaouthia*, NKA), Russell’s viper (*Daboia russelii*, DRU), and the common tiger snake (*Notechis scutatus*, NSC). No significant differences were detected in short interspersed nuclear elements (SINEs) (*p* = 0.6043) and DNA transposons (*p* = 0.9614). Significant differences were detected in long terminal repeat (LTR) elements (*p* = 0.0253), long interspersed nuclear elements (LINEs) (*p* = 0.0037), and total interspersed repeats (*p* = 0.0018). Data are shown as mean ± standard deviation. The box includes 50% of the data, and the whiskers reach the highest and lowest values within 95% of the distribution. Open circles represent single values outside 95% of the distribution. Degrees of statistical significance were represented as * for *p* ≤ 0.05, and ** for *p* ≤ 0.01.

Sixteen BACs mapped on snake W sex chromosomes were screened for DNA repeat abundance and 7.91% masked areas were recorded by RepeatMasker. This represented a total length spanning 213,424 base pairs (bp) of 2,698,299 bp of these sequences being annotated as repeat elements. Apart from microsatellite repeat motifs, the majority of these repeats were identified as retroelements such as long interspersed nuclear elements (LINEs) (6.18 ± 3.61%) and short interspersed nuclear elements (SINEs) (0.10 ± 0.12%) (Figure 4 and Supplementary Figure S1). The BAC sequences also contained certain DNA transposons such as hobo-activator, Tc1-IS360-Pogo, and Tourist/Harbinger. For BACs mapped on the autosomes of snakes, 6.04% masked areas were recorded for DNA repeat abundance. This represented a total length spanning 351,270 base pairs (bp) of 5,811,518 bp of these sequences. Apart from microsatellite repeat motifs, the majority of these repeats were identified as retroelements, such as LINEs (2.93 ± 2.36%) and SINEs (0.12 ± 0.12%) (Figure 4 and Supplementary Figure S2). The BAC sequences contained certain DNA transposons such as hobo-activator. MSAs of all candidate BACs mapped on snake W sex chromosomes showed no conserved sequences/elements (Supplementary Figure S3).

## Discussion

Sex chromosomes have important evolutionary consequences in speciation, sexual dimorphism, and sexual antagonism (Rice et al., 2008; Dean and Mank, 2014; Ezaz et al., 2017). Amniotes are well known for their diverse modes of sex determination mechanisms and sex chromosomes that exhibit extraordinary variability as either homomorphic or heteromorphic structures (Graves, 2016; Ezaz et al., 2017). Karyotypes of birds and snakes are highly conserved in the Z sex chromosome of each lineage, and show diverse patterns of W sex chromosome degeneration in different clades. Conversely, most mammals show X sex chromosome conservation and variable Y sex chromosome differentiation (Waters et al., 2007; Bachtrog, 2013). By contrast, in other SRs, rapid evolution of non-homologous sex chromosomes has been reported within even closely related species (Pokorná and Kratochvíl, 2009; Kawagoshi et al., 2014). This suggests that sex chromosomes and sex determination systems evolved independently in different amniote lineages.

### Prescriptive Analysis of Ancestral Amniote Super-Sex Chromosome

**FIGURE 5:**
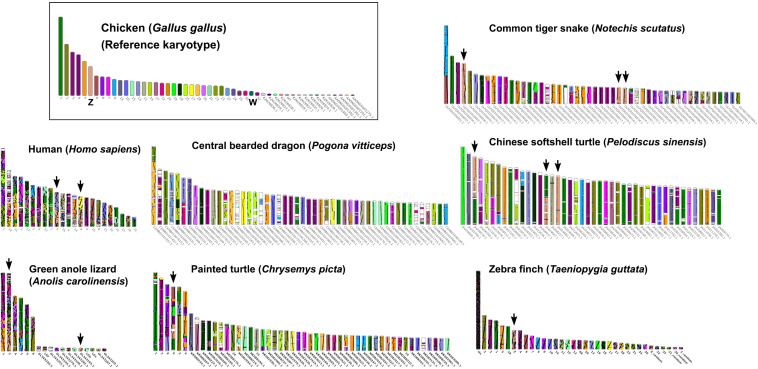
Karyotype evolution of representative amniotes. A multi-karyotype view is shown for the reference chicken (*Gallus gallus*) genome. Reference chromosomes of the chicken share genomic sequences with common tiger snake (*Notechis scutatus*), human (*Homo sapiens*), central bearded dragon (*Pogona vitticeps*), Chinese softshell turtle (*Pelodiscus sinensis*), green anole lizard (*Anolis carolinensis*), painted turtle (*Chrysemys picta*), and zebra finch (*Taeniopygia guttata*) distinguished with different colors for homologs. Black arrows highlight homology with the chicken Z chromosome. The common tiger snake karyotype schematic shows the 50 longest scaffolds.

Multi-karyotypes with whole genome comparison of amniotes under simulation of the Genomicus and Synteny portals showed sex chromosomal linkage homologies among amniotes (Lee et al., 2016; Nguyen et al., 2018) (Figure 5). Many sex chromosomes in amniotes show a high degree of sequence similarity. Moreover, the combination of whole genome sequence analyses and comparative gene mapping (chromosome mapping via a cytogenetic technique), also known as chromosomics, revealed that these sex chromosomal linkage homologies involve genomic regions orthologous to SR2 and the snake W sex chromosome. This supports the hypothesis that both sex chromosomes are a major part of a larger ancestral amniote super-sex chromosome (Ezaz et al., 2017; Singchat et al., 2018, 2020; Deakin et al., 2019; Matsubara et al., 2019; Ahmad et al., 2020). Perhaps the 1967 hypothesis of Ohno might be partially correct. Several microsatellite repeat motifs with chromosome mapping show repeat sequences shared between the sex chromosomes of several amniotes (Matsubara et al., 2013, 2015a,b, 2016b; Rovatsos et al., 2015; Augstenová et al., 2018; Perry et al., 2018; Singchat et al., 2018). Pythons (primitive snakes), as a group with homomorphic sex chromosomes in the basal position of snake phylogeny do not show any accumulation of repeats, whereas the degenerated W sex chromosomes in many advanced snakes exhibit a massive accumulation of repeats with C-positive heterochromatin (Mengden, 1981; O’Meally et al., 2010; Matsubara et al., 2015a, 2016b; Augstenová et al., 2018; Singchat et al., 2018; Thongchum et al., 2019). The large W sex chromosome of NSC is composed almost entirely of repeats that are partially shared with the chicken W sex chromosome (O’Meally et al., 2010). Unlike the idea of accidental random homology as previously discussed (Singchat et al., 2018, 2020; Matsubara et al., 2019), 16 BACs derived from GGA1, GGA4, GGA9, GGAZ, TGU1B, TGU9, TGU13, TGU17, TGU23, TGU27, and TGUZ, which showed partial linkage homologies with various amniote sex chromosomes, were mapped on DRUW and/or NSCW in addition to NKAW (Singchat et al., 2018). The results suggest that these BACs contain a common genomic region such as repeat sequences, also shared with DRUW and NSCW. Notably, two GGAZ BACs (CH261-133M4 and TGMCBA-270I9) were expected to be located on snake chromosome 2 (Matsubara et al., 2006; Singchat et al., 2018). However, GGAZ BAC (CH261-133M4) was located on NKA2q, NSC2p, and NSCW, whereas GGAZ BAC (TGMCBA-270I9) was localized to NKA2q, NSCW, and DRUW, suggesting an association between chromosome 2 and snake W sex chromosome. Using the combination of a similar set of BAC chromosome mapping, no similar hybridization patterns were found in *Iguana iguana*, *Calotes versicolor*, and *Varanus salvator macromaculatus* (Singchat et al., 2020). Partial linkage homology of GGA1, corresponding to the micro-Z chromosomes of *Varanus komodoensis* was also observed in NKA2 and VSA microchromosomes, and partial homology of GGA15 was observed in CVE2 homologous to SR2. These observations of hybridization patterns of all BACs mapped on snake W sex chromosomes confirm that a large ancestral super-sex chromosome has robust correlation between SR2 and snake W sex chromosomes (Singchat et al., 2018, 2020). A series of fusion–fission events between macrochromosomes or other microchromosomes resulted in diversified karyotypes among amniotes (Olmo and Signorino, 2005; Matsubara et al., 2006, 2012, 2019; Srikulnath et al., 2009a,b, 2011, 2013, 2014, 2015, 2019; Alföldi et al., 2011; Singchat et al., 2018). These results collectively suggest that multiple fission events occurred in ancestral amniote super-sex chromosomes involving SR2 and the snake W sex chromosome, which resulted in a variety of sex chromosomal linkages in different lineages (Figure 3).

Morphological variation that occurs specifically in sex chromosomes of different amniote lineages is probably caused by chromosomal rearrangements involving different genes on contrasting chromosomal regions (Rieseberg, 2001; Faria and Navarro, 2010). This in turn leads to diversity in autosome number, sex chromosome system, and sex determination genes, and probably partially contributes to species diversity (Charlesworth et al., 2005; Pennell et al., 2015). There is an ongoing debate as to whether certain chromosomes are more likely to become sex chromosomes than others (Johnson and Lachance, 2012; Landeen and Presgraves, 2013). The testis-determining gene *DMRT1* is located on sex chromosomes in several amniotes (Eggers and Sinclair, 2012; Kopp, 2012; Webster et al., 2017). Banded krait minor satellite (*Bkm*) repeats consist of tandem arrays of two tetranucleotides, GATA and GACA (Singh et al., 1976; Epplen et al., 1982). *Bkm*-related repeats are also accumulated on heterogametic sex chromosomes in many amniotes, although they are also found on autosomes (Jones and Singh, 1985; Nanda et al., 1991). *Bkm*-related repeats are functional, playing a role in the transcriptional activation of heterochromatin on sex chromosomes (Singh et al., 1976), and this is more likely to reflect a convergent evolution of degeneration. It seems that historical contingency, rather than the characteristics of particular sequences, plays a major role in the determination of sex chromosomes in a particular lineage. Our results show several BACs in relation to snake W sex chromosome and SR2. To determine conserved segments predictively, common genomic elements such as repeat sequences or non-coding segments/elements might result in hybridization of the same BACs onto multiple different chromosome pairs, suggesting the contribution of transitions to diverse modes and mechanisms of sex chromosomes across amniotes. Therefore, are any specific genomic segments/elements shared among unrelated sex chromosomal linkages in amniotes, which drive sex chromosome differentiation after chromosomal rearrangement in each lineage?

### Conserved Elements Among Segmental Linkage Homology

Potential specialization of functional genes, or conserved elements associated with sex development and sex determination in different genomic compartments, remains a fundamental biological question concerning ancestral amniote super-sex chromosomes. Prediction of genomic content of all BACs mapped on snake W sex chromosomes has revealed that most were LINE and SINE retrotransposons, in addition to the microsatellite repeat motifs found in our previous study (Singchat et al., 2018, 2020). This result leads us to predict that amplification of LINE and SINE might have occurred during the evolution of snake W chromosomes. This aligns with the presence of transposon amplification on snake sex chromosomes and other amniotes (Kraemer and Schmidt, 1993; Erlandsson et al., 2000; Peichel et al., 2004; Rovatsos et al., 2015; Śliwińska et al., 2016; Dechaud et al., 2019). Discernment of the causes and consequences of genome compartmentalization such as transposon amplification is key to understanding sex chromosome differentiation. This might influence chromosome structure and number and/or the linkage homology of the genes they contain. PBI-*Dde*I satellite DNA repeats are observed in centromeric regions of all chromosomes in python (*P. bivittatus*) with a large copy number variation (Matsubara et al., 2015a), whereas high amplification of satellite DNA is found on the Siamese cobra W sex chromosome (Thongchum et al., 2019). This suggests that these occurrences are associated with functionally evolutionary transitions in sex chromosome differentiation. However, there is a lack of evidence to prove that LINE and SINE retrotransposons impact sex chromosome differentiation. Alternatively, the presence and absence of hybridization signals on different species from mapped BACs result from the amplification of repeat sequences that occur independently in each lineage and at different rates. The same BACs have been used to perform chromosome mapping in other amniotes that did not show hybridization signals, such as repeat sequences or accumulation of BACs on sex chromosomes (Damas et al., 2017; O’Connor et al., 2018a,b; Singchat et al., 2018, 2020).

To investigate other conserved elements in addition to repeats, MSAs of all BAC sequences have been conducted, but no significant elements have been observed. Molecular cloning and functional genomic study of this gene are necessary to investigate and annotate the key elements. However, in any explanation of the evolutionary origin of an ancestral super-sex chromosome, it is possible that sex-linked conserved elements are shared across sex chromosomes of amniotes. The amniote super-sex chromosome might have shared certain *de novo* sequences that remain hitherto unknown. To test this hypothesis, we recommend that complete sequences of many amniote sex chromosomes should be thoroughly explored to investigate any putative sex-linked orthologs. This can be achieved by overcoming the challenges and difficulties of assembling sex chromosomes through the availability of high quality genome assemblies and annotation of amniotes.

Combining our data with previously published work indicates that unrelated sex chromosomal linkage homologies in amniotes were shared with SR2 and snake W sex chromosomes. However, we found no putative conserved element identification shared among unrelated sex chromosomal linkage homologies. Functional genes and clusters of retrotransposons were identified, which might drive functionally convergent evolution of sex chromosomes, such as starters for heterochromatinization. Without extensive characterization of the shared repeat by sequence analysis and functional genomics with large multiple species, it is difficult to indicate its origins and possible functional roles. Therefore, comparative genomics of sex chromosomes must be conducted based on good quality genome assemblies in amniotes. Our results support the concept of sex chromosome evolution as a colorful myriad of situations and trajectories involving many interacting processes.

## Data Availability Statement

All datasets presented in this study are included in the article/Supplementary Material.

## Ethics Statement

Ethical review and approval was not required for the animal study because cell suspensions of two female species [Russell’s viper (DRU) and the common tiger snake (NSC)] were provided by Malcolm Ferguson-Smith (Cell Collection, Department of Veterinary Medicine, University of Cambridge, United Kingdom) in November 2019. We did not perform any live animals in this study.

## Author Contributions

WS and KS drafted the manuscript. WS, SA, RO’C, DG, and KS conceived the ideas and designed the methodology. WS, SA, and KS conducted the lab work and participated in the data analysis. All authors reviewed the data and the manuscript, and gave final approval for publication.

## Conflict of Interest

The authors declare that the research was conducted in the absence of any commercial or financial relationships that could be construed as a potential conflict of interest.

## Footnotes

1 http://www.geneious.com/

2 https://www.ensembl.org/

3 https://www.ncbi.nlm.nih.gov/genome/annotation_euk/Gallus_gallus/104/

4 https://www.ncbi.nlm.nih.gov/genome/annotation_euk/Taeniopygia_guttata/103/

## Supplementary Material

FIGURE S1 Total number of repetitive elements with total length (base pairs) in the chicken and zebra finch BACs, mapped on the W sex chromosome in Siamese cobra, Russell’s viper, and the common tiger snake.

FIGURE S2 Total number of repetitive elements with total length (base pairs) in the chicken and zebra finch BACs, mapped on autosomes in Siamese cobra, Russell’s viper, and the common tiger snake.

FIGURE S3 Multiple sequence alignment to identify any possible conserved sequence of 16 BACs mapped on the W sex chromosome of snake. A representative region is shown confirming distinct sequences that are non-homologous to each other.
