# Supplementary figures and images for: Partial Amniote Sex Chromosomal Linkage Homologies Shared on Snake W Sex Chromosomes Support the Ancestral Super-Sex Chromosome Evolution in Amniotes
Source: Front Genet. 2020 Aug 18;11:948. doi: 10.3389/fgene.2020.00948 (PMC7461878; doi:10.3389/fgene.2020.00948)

## Supplementary Figure

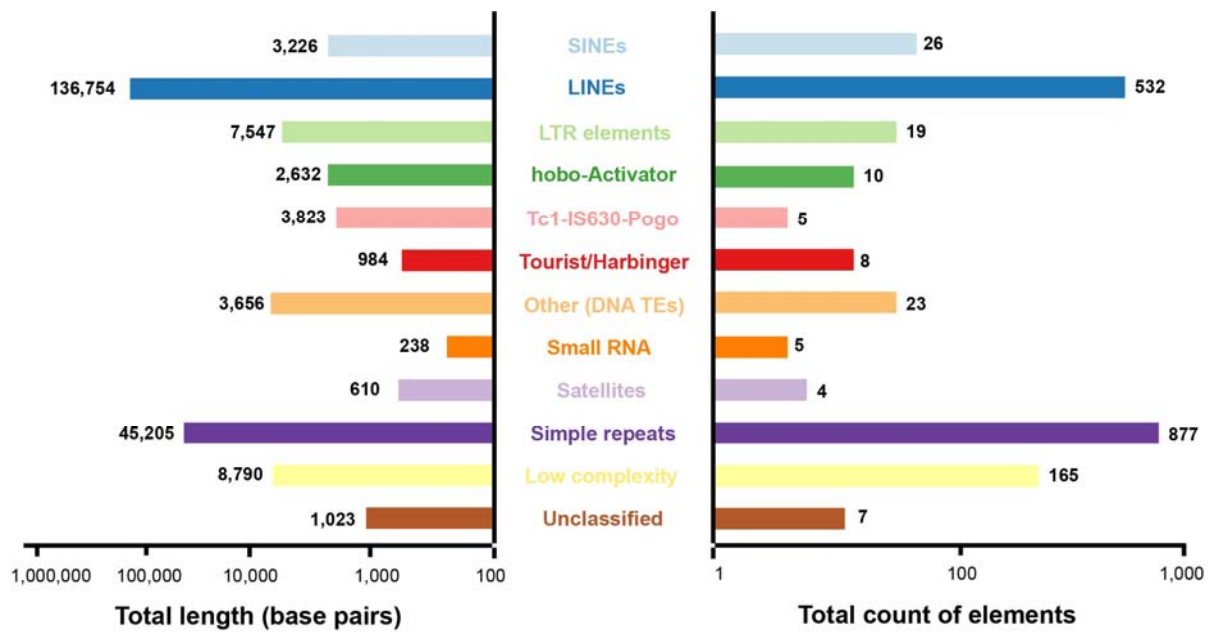

FIGURE S1 | (Singchat et al.)

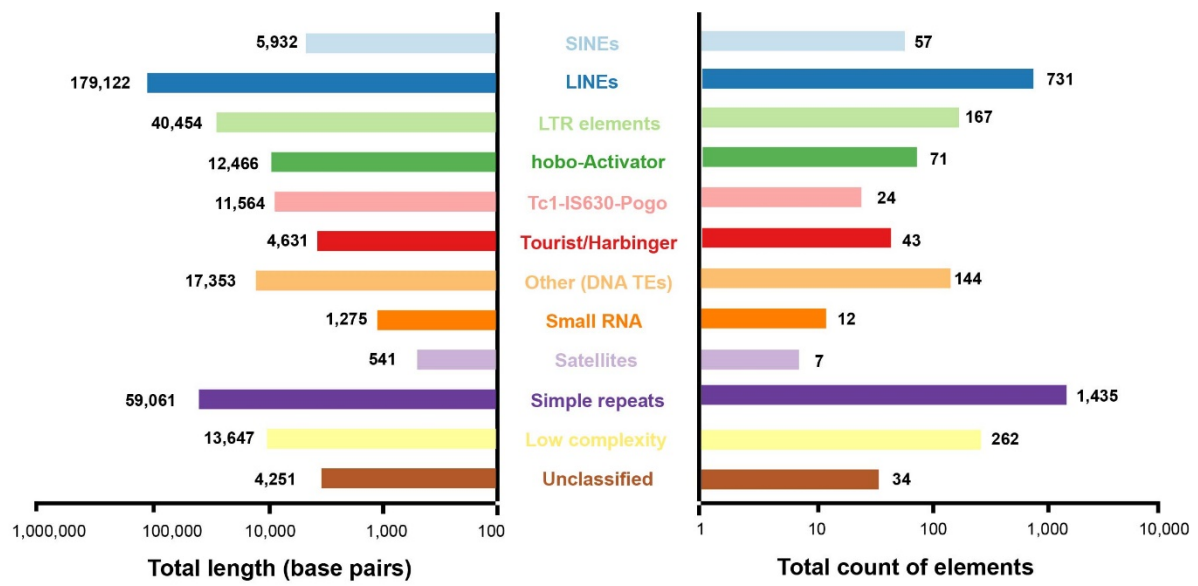

**FIGURE S2** | (Singchat et al.)

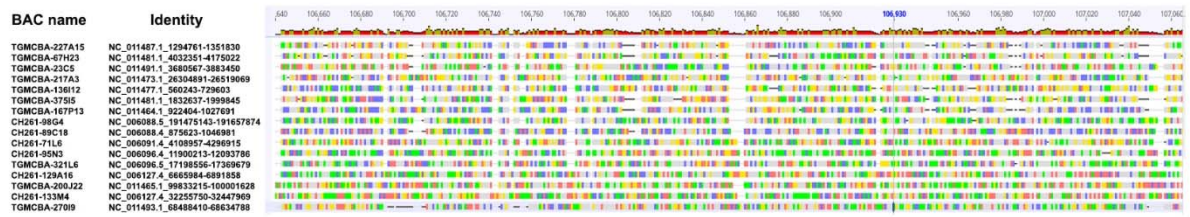

FIGURE S3 | (Singchat et al.)

Supplement: FIGURE S1 — Total number of repetitive elements with total length (base pairs) in the chicken and zebra finch BACs, mapped on the W sex chromosome in Siamese cobra, Russell’s viper, and the common tiger snake. [file Data_Sheet_1.pdf]
